# The MAGENTA Model for Individual Prediction of In-Hospital Mortality in Chronic Obstructive Pulmonary Disease With Acute Exacerbation: An External Validation Study

**DOI:** 10.14740/jocmr6512

**Published:** 2026-03-26

**Authors:** Thotsaporn Morasert, Chayatorn Tanksinmankhong, Pichaya Tantiyavarong, Phanu Prasankiattirach, Pakpoom Wongyikul, Phichayut Phinyo

**Affiliations:** a Department of Clinical Epidemiology, Faculty of Medicine, Thammasat University, Pathum Thani 10120, Thailand; b Department of Internal Medicine, Suratthani Hospital, Surat Thani 84000, Thailand; c Centre for Clinical Epidemiology and Clinical Statistics, Faculty of Medicine, Chiang Mai University, Chiang Mai 50200, Thailand; d Department of Biomedical Informatics and Clinical Epidemiology (BioCE), Faculty of Medicine, Chiang Mai University, Chiang Mai 50200, Thailand

**Keywords:** COPD exacerbation, In-hospital mortality, MAGENTA model, External validation

## Abstract

**Background:**

The MAGENTA score identifies acute exacerbation of chronic obstructive pulmonary disease (AECOPD) patients at high risk of in-hospital mortality to guide monitoring and treatment, yet its generalizability requires confirmation. This study aimed to externally validate the performance of the MAGENTA model.

**Methods:**

We conducted a temporal external validation using retrospective data from 938 admission records of patients hospitalized in general wards and intensive care units at a tertiary center in Thailand (2018–2019). The model, which utilizes mean arterial pressure, age, blood urea nitrogen, endotracheal intubation, sodium, temperature, and albumin, was evaluated regarding all-cause in-hospital mortality.

**Results:**

The validation cohort had an 11.2% mortality rate with moderate case-mix differences compared to the development set. The model demonstrated acceptable discrimination with an area under the curve (AUC) of 0.75 (95% confidence interval (CI), 0.70–0.80), though lower than the AUC in the original derivation. Calibration analysis revealed systematic overprediction (expected-to-observed (E:O) ratio of 1.335) and overfitting (slope of 0.536), particularly when the predicted risk exceeded 20%. Importantly, recalibration of the intercept and slope substantially improved the agreement between predicted and observed risks.

**Conclusions:**

While the MAGENTA model offers acceptable discriminative ability for stratifying AECOPD mortality risk, local recalibration is recommended to address overestimation in high-risk patients.

## Introduction

Acute exacerbation of chronic obstructive pulmonary disease (AECOPD) is associated with increased mortality, particularly in patients who require hospitalization. Thirty percent of AECOPD require hospitalization [1, 2], which results in an even higher mortality risk [3]. The in-hospital mortality varied among different studies, ranging from 2.5% to 25% [4, 5]. Furthermore, 22% of patients who survived to hospital discharge died within 1 year after discharge [6].

There are several prognostic factors associated with short-term mortality among hospitalized AECOPD patients, including age, male sex, body mass index (BMI), current smoking and comorbidities (cardiac failure, chronic renal failure, smoking, diabetes, ischemic heart disease), long-term oxygen therapy, lower limb edema, chronic steroid use, disease-specific severity features (forced expiratory volume in 1 s (FEV1), Global Initiative for Chronic Obstructive Lung Disease (GOLD) stage 4, cor pulmonale), and laboratory parameters (acidemia, PCO_2_ and PO_2_ on admission) [7]. International chronic obstructive pulmonary disease (COPD) guidelines also recommend clinical prediction tools or scoring systems to help clinicians triage the level of care [8, 9].

Accordingly, developing an accurate prediction model for individual in-hospital mortality in COPD with acute exacerbation can provide valuable information to clinicians, patients, and families. Although the DECAF score has demonstrated strong performance [10], implementation may be constrained in some settings because certain predictors (e.g., arterial blood gas) are not consistently available in routine care. Blood eosinophil count (BEC) is increasingly recognized as a useful biomarker in AECOPD, particularly for identifying eosinophilic exacerbations and informing the likelihood of benefit from systemic corticosteroids in selected patients [11, 12]. However, the interpretation and routine incorporation of BEC into bedside risk scores may be challenging in resource-limited or high–infectious-burden contexts, where eosinophil levels can be influenced by comorbid allergic disease, parasitic infections, and other causes, and where testing practices and timing may vary [13].

Recently, a novel predictive model called the MAGENTA score has been developed for individual prediction of in-hospital mortality in patients with AECOPD in resource-limited countries [14]. The MAGENTA score is based on clinical and laboratory parameters. This score included mean arterial pressure (MAP), age, blood urea nitrogen (BUN), endotracheal intubation, sodium (Na), body temperature, and serum albumin. The area under the curve (AUC) for the prediction of in-hospital mortality among AECOPD patients was 0.82 (95% confidence interval (CI), 0.77–0.86). External validation is necessary to ensure the robustness of the predictive performance and generalizability of the MAGENTA score in AECOPD patients. In addition, the external validation study results will provide further evidence of the MAGENTA score’s accuracy and reliability, increasing its potential to be widely adopted by healthcare professionals to guide clinical decision-making and improve patient outcomes.

## Materials and Methods

We conducted a validation study for a developed prediction model and reported the study, following the Transparent Reporting of a Multivariable Prediction Model for Individual Prognosis or Diagnosis (TRIPOD): The TRIPOD statement [15].

### Source of data

This external validation study was derived from a retrospective cohort study. The validation data were obtained from admission records of AECOPD patients during October 2018–September 2019 after the development dataset of the previously published MAGENTA model [14]. The validation dataset was collected during a later time frame and was completely independent from the original development cohort, thereby representing a temporal external validation conducted within the same institution. The ethical committee of Suratthani Hospital, Thailand, approved the study protocol (protocol code: COA 004/2566, January 16, 2023). This study was conducted in accordance with the Declaration of Helsinki.

### Participants

The study participants were COPD patients who presented with acute exacerbation and required hospitalization in a university-affiliated tertiary care center, Suratthani Hospital, Thailand. This study included all admissions to the general medical ward and the medical intensive care unit (ICU). The diagnosis of AECOPD was based on the “principal diagnosis” by the International Statistical Classification of Diseases and Related Health Problems, 10th Revision (ICD-10) codes J44.0, J44.1, and J44.9 in the discharge summary. The exclusion criteria were: 1) age < 40 years; and 2) spirometry results incompatible with COPD (FEV1/forced vital capacity (FVC) ratio > 0.7), when spirometry data were available. In cases of missing spirometry result, the diagnosis of AECOPD was based on the ICD-10 codes at discharge. The unit of observation in this study was each hospitalized admission for each COPD patient. In addition, the data were collected in multiple records for the patient with recurrent admissions during the study period.

### Data collection

All data were collected and managed using REDCap^®^ electronic data capture tools hosted at Suratthani Hospital Medical Education Center. The demographic data, underlying diseases, COPD status, medications, and initial admission parameters were collected from the admission records. Spirometry results were collected from reports within 1 year before index admission. Laboratory results from the initial 24 h of admission were collected through electronic laboratory records. In addition, the presence of pulmonary consolidation on chest radiography, indicating pneumonia, was obtained from the physician progress notes. Data sources and roles of variables were demonstrated here (Supplementary Material 1, jocmr.elmerjournals.com).

### Outcome

All patient admission records were separated into two outcomes: non-surviving admissions and surviving admissions based on the survival status of the patient in the admission discharge summary.

### Predictors

The MAGENTA model consists of seven commonly available clinical parameters: age, body temperature, MAP, the requirement of endotracheal intubation, serum sodium (SNa), BUN, and serum albumin. In addition, a detailed equation for predicting the probability of in-hospital mortality of hospitalized AECOPD was published [14]. The online web application of the MAGENTA model is available [16]. All parameters were collected at the initial admission and the first 24 h for investigations (Supplementary Material 1, jocmr.elmerjournals.com).

### Sample size

The minimum sample size for validating a clinical prediction model was determined using the third criterion proposed by Riley et al [17]. This approach requires the expected discriminative ability (AUC), the outcome prevalence, and the acceptable error margin of the AUC CI. For an anticipated AUC of 0.82, an error margin of 0.1, and an expected in-hospital mortality prevalence of 11% from the development data published, the required sample size was 700 patients with 77 mortality events.

### Missing data

All missing data were handled under the assumption of missing at random (MAR) and were imputed using multiple imputation with chained equations (MICE) [18]. Age, gender, MAP, radiographic consolidation, requirement of endotracheal intubation, BUN, serum creatinine (SCr), and survival status (the study endpoint) were used as auxiliary variables in predictive mean matching (PMM) methods with K-nearest neighbor, where k = 10.

### Statistical analysis

We compared the clinical characteristics between non-surviving and surviving hospitalized AECOPD patients. Categorical variables were presented as percentages and compared using Fisher’s exact test. Continuous variables were presented as mean ± standard deviation (SD) and compared using a two-sample *t*-test. Non-parametric continuous variables were presented as the median with interquartile range (IQR) and compared using the Wilcoxon Rank Sum (Mann–Whitney) test. All proportions and two-sided P values were calculated among non-missing data.

We followed a three-step framework for external validation, recently proposed by Debray et al. First, we assessed the relatedness (similar or different) between the development and validation datasets by estimating the discriminative ability of the membership model [19]. This model is a binary logistic model, with the dataset (either development or validation) as the dependent variable and predictors of the MAGENTA model and in-hospital mortality as independent variables. The discriminative ability of the model was expressed using AUC. A high AUC would indicate strong differences between the datasets in terms of predictors and outcome, whereas a low AUC (close to 0.5) would suggest greater similarity. We also estimated the linear predictor (LP) of the MAGENTA model and its SD for both datasets, which were used to reflect the distribution of case mix in each dataset. Independent *t*-test and variance-comparison test were used to compare the mean and SD of the LP, respectively.

Second, we evaluated external model performance in two aspects: model discrimination and model calibration. We used the MAGENTA model for each admission to predict the probability of in-hospital mortality. The discriminative ability of the model’s predicted probability was quantified using AUC. According to the classification proposed by Hosmer et al (2000), an AUC of 0.70 to 0.80 represents acceptable discrimination, 0.80 to 0.90 represents excellent discrimination, and > 0.90 represents outstanding discrimination [20]. For model calibration, the expected-to-observed (E:O) ratio, calibration-in-the-large (CITL), and calibration slope were estimated. The E:O ratio compares the number of predicted to observed events; a ratio of 1 indicates perfect agreement, values > 1 suggest overprediction, and < 1 suggest underprediction. CITL reflects whether predicted risks are systematically too high or too low: a CITL of 0 is ideal, negative values indicate overprediction, and positive values indicate underprediction. The calibration slope assesses whether predictions are proportionate with observed risk over the entire range of predicted risk; a slope of 1 is perfect, < 1 means the model overfits (too much variation in prediction), and > 1 means it underfits (predicted risks do not vary enough) [19, 21].

Third, we interpreted the model validation results based on the first and second steps. In case of poor model performance, updating methods will be applied depending on the aspects of performance, such as recalibration of model intercept, model slope, or reweighting the predictor coefficients.

## Results

### Admission and patients’ characteristics

**Figure 1:**
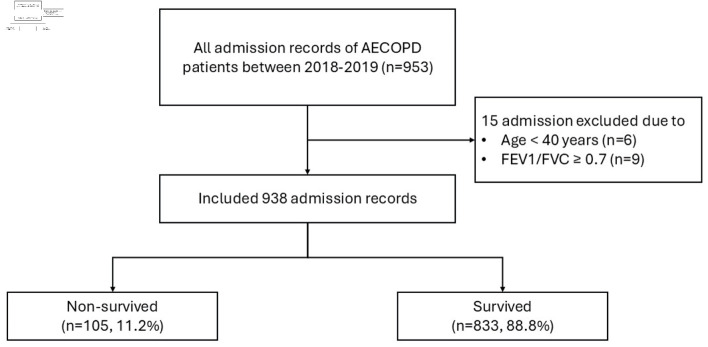
Study flow diagram of the patient cohort. AECOPD: acute exacerbation of chronic obstructive pulmonary disease; FEV1: forced expiratory volume in 1 second; FVC: forced vital capacity.

A total of 953 admissions of AECOPD were retrieved. Fifteen admissions were excluded (age < 40 years: six admissions, FEV1/FVC > 0.7: nine admissions). Finally, the external validation dataset included 938 admission records of 668 AECOPD patients. Of these numbers, there were 105 (11.2%) non-survived admissions (Fig. 1), which is close to the development dataset (10.9%) [12]. The albumin level information was missing for 436 admissions (46.5%) in the original version of the validation cohort. The comparisons of characteristics, clinical parameters and laboratory investigation during admission among survived and non-survived admissions of the validation dataset are shown in Tables 1 and 2. The patients were predominantly male (86%), with a mean age of 74.8 (± 11.3) years. Eighty-six per cent of all patients were smokers (ex-smokers: 70%, active smokers: 16%). Only 23% of the patients had spirometry results. The mean of FEV1/FVC was 0.51 (± 0.10), and the mean FEV1 was 44% (± 17%). Compared with patients in the development dataset, the clinical characteristics of those in validation datasets were similar, except there were no significant differences in age, initial body temperature and serum sodium among the survived and non-survived groups in the validation dataset (Table 3).

**Table 1:** Characteristics of Survived and Non-Survived Admissions With AECOPD (N = 938 Admissions)

Characteristics	Missing data, n (%)	Non-survived admissions, n (%)	Survived admissions, n (%)	P value
Total		105 (11.2)	833 (88.8)	
Male	0 (0)	87 (82.9)	718 (86.2)	0.370
Age, years, mean ± SD	0 (0)	76.7 ± 10.1	74.6 ± 11.4	0.072
Body mass index, kg/m^2^, mean ± SD	121 (12.9)	12.0 ± 8.0	16.6 ± 7.4	< 0.001
Smoking status				0.110
Never smoker	22 (2.4)	19 (18.1)	109 (13.1)	
Ex-smoker	22 (2.4)	64 (61.0)	580 (69.6)	
Active smoker	22 (2.4)	21 (20.0)	123 (14.8)	
Number of cigarettes smoked				
Pack-year, median (IQR)	212 (22.6)	30.0 (10.0, 40.0)	25.0 (15.0, 40.0)	0.690
Underlying diseases				
Presence (any)	0 (0)	90 (85.7)	697 (83.7)	0.670
Hypertension	0 (0)	50 (47.6)	394 (47.3)	1.000
Diabetes mellitus	0 (0)	22 (21.0)	133 (16.0)	0.210
Ischemic heart disease	0 (0)	12 (11.4)	70 (8.4)	0.280
Atrial fibrillation	0 (0)	13 (12.4)	52 (6.2)	0.038
Left ventricular dysfunction	0 (0)	1 (1.0)	10 (1.2)	1.000
Chronic kidney disease	0 (0)	14 (13.3)	102 (12.2)	0.750
Cerebrovascular disease	0 (0)	8 (7.6)	82 (9.8)	0.600
Cognitive impairment	0 (0)	3 (2.9)	15 (1.8)	0.440
COPD status				
Spirometry performed, n (%)	719 (76.7)	10 (9.5)	209 (25.1)	< 0.001
FEV1/FVC ratio, mean ± SD	719 (76.7)	0.5 ± 0.1	0.5 ± 0.1	0.980
FEV1, % predicted, mean ± SD	719 (76.7)	35.4 ± 11.8	44.7 ± 17.3	0.096
FVC, % predicted, mean ± SD	719 (76.7)	53.3 ± 9.6	66.9 ± 18.2	0.021
Long-term oxygen therapy	0 (0)	3 (2.9)	32 (3.8)	0.790
Cor pulmonale	805 (85.8)	3 (2.9)	25 (3.0)	1.000
Inhaled controller medications				
Presence (any)	0 (0)	76 (72.4)	614 (73.7)	0.810
Salmeterol/fluticasone	0 (0)	64 (61.0)	533 (64.0)	0.590
Formoterol/budesonide	0 (0)	1 (1.0)	21 (2.5)	0.500
Tiotropium	0 (0)	18 (17.1)	210 (25.2)	0.071
Budesonide	0 (0)	4 (3.8)	38 (4.6)	1.000
Influenza vaccination	0 (0)	5 (4.8)	56 (6.7)	0.530

AECOPD: acute exacerbation of chronic obstructive pulmonary disease; FEV1: forced expiratory volume in 1 second; FVC: forced vital capacity; IQR: interquartile range; SD: standard deviation.

**Table 2:** Clinical Parameters During Admission of Survived and Non-Survived Admissions With AECOPD (N = 938 Admissions)

Clinical parameters	Missing data, n (%)	Non-survived admissions, n (%) (n = 105)	Survived admissions, n (%) (n = 833)	P value
Initial vital signs				
Body temperature, °C, mean ± SD	0 (0)	37.5 ± 0.9	37.4 ± 1.0	0.220
Heart rate, beats/min, mean ± SD	0 (0)	111.6 ± 30.7	111.4 ± 22.4	0.930
Systolic BP, mm Hg, mean ± SD	0 (0)	129.6 ± 35.9	142.9 ± 29.5	< 0.001
Diastolic BP, mm Hg, mean ± SD	0 (0)	76.7 ± 20.4	84.4 ± 17.3	< 0.001
Mean arterial pressure, mm Hg, mean ± SD	0 (0)	94.4 ± 24.4	103.9 ± 19.8	< 0.001
Respiratory rate, breaths/min, mean ± SD	2 (0.2)	34.3 ± 10.2	34.7 ± 7.4	0.660
Required endotracheal intubation, n (%)	2 (0.2)	100 (95.2)	583 (70.0)	< 0.001
Radiographic consolidation	0 (0)	59 (56.2)	174 (20.9)	< 0.001
Laboratory investigations				
Sodium, mmol/L, mean ± SD	8 (0.9)	138.3 ± 5.8	138.6 ± 5.7	0.650
Bicarbonate, mmol/L, mean ± SD	8 (0.9)	23.7 ± 6.8	24.2 ± 5.2	0.380
Blood urea nitrogen, mg/dL, median (IQR)	8 (0.9)	22.5 (14.0, 35.0)	15.0 (11.0, 21.0)	< 0.001
Serum creatinine, mg/dL, median (IQR)	2 (0.2)	1.1 (0.8, 1.5)	0.9 (0.7, 1.2)	< 0.001
Serum albumin, g/dL, mean ± SD	436 (46.5)	3.2 ± 0.6	3.8 ± 0.6	< 0.001
Hemoglobin, g/dL, mean ± SD	5 (0.5)	11.9 ± 2.2	12.6 ± 2.0	< 0.001
WBC, cells/mm^3^, median (IQR)	5 (0.5)	14,000 (9,400, 19,700)	12,980 (9,850, 16,550)	0.138
Neutrophil percent, mean ± SD	5 (0.5)	83.0 ± 14.0	84.5 ± 12.7	0.240
Eosinophil count, cells/mm^3^, median (IQR)	5 (0.5)	0.0 (0.0, 101.6)	14.2 (0, 194.8)	0.020
Length of hospital stay, days, median (IQR)	0 (0)	11.0 (3.0, 18.0)	3.0 (2.0, 6.0)	< 0.001

AECOPD: acute exacerbation of chronic obstructive pulmonary disease; BP: blood pressure; IQR: interquartile range; SD: standard deviation; WBC: white blood cell count.

**Table 3:** Clinical Characteristics of the Patients in the Validation Dataset and the Development Dataset

Clinical characteristic	Validation dataset (n = 938)				Development dataset (n = 923)			
	Missing values	Non-survived admissions (n = 105)	Survived admissions (n = 833)	P value	Missing values	Non-survived admissions (n = 101)	Survived admissions (n = 822)	P value
	n (%)	Mean ± SD	Mean ± SD		n (%)	Mean ± SD	Mean ± SD	
Demographic characteristics								
Age, years	0 (0)	76.7 ± 10.1	74.6 ± 11.4	0.072	0 (0)	76.8 ± 11.0	74.1 ± 11.0	0.020
Initial assessments								
BT, °C	0 (0)	37.5 ± 0.9	37.4 ± 1.0	0.220	1 (0.1)	37.4 ± 0.9	37.1 ± 0.6	< 0.001
MAP, mm Hg	0 (0)	94.4 ± 24.4	103.9 ± 19.8	< 0.001	0 (0)	90.3 ± 20.3	98.3 ± 15.4	< 0.001
Required endotracheal intubation, n (%)	0 (0)	100 (95.2)	583 (70.0)	< 0.001	0 (0)	96 (95.1)	561 (68.3)	< 0.001
Initial investigations								
Radiographic consolidation, n (%)	2 (0.2)	59 (56.2)	174 (20.9)	< 0.001	0 (0)	65 (64.4)	304 (37.0)	< 0.001
Complete blood count								
WBC count, cells/mm^3^	5 (0.5)	16,065 ± 10,697	13,767 ± 6,106	0.001	1 (0.1)	15,296 ± 6,978	13,738 ± 6,083	0.017
Eosinophil count, cells/mm^3^, median (IQR)	5 (0.5)	0 (0, 102)	14.2 (0, 195)	0.020	1 (0.1)	9.5 (0, 172)	40.8 (0, 228)	0.010
Blood chemistry								
Sodium, mmol/L	8 (0.9)	138.3 ± 5.8	138.6 ± 5.7	0.650	4 (0.4)	137.5 ± 7.6	138.8 ± 4.6	0.013
BUN, mg/dL, median (IQR)	8 (0.9)	22.5 (14.0, 35.0)	15.0 (11.0, 21.0)	< 0.001	0 (0)	21 (14, 32)	15 (11, 21)	< 0.001
SCr, mg/dL, median (IQR)	2 (0.2)	1.1 (0.8, 1.5)	0.9 (0.7, 1.2)	< 0.001	0 (0)	1.1 (0.8, 1.5)	0.9 (0.8, 1.2)	0.002
Serum albumin, g/dL	436 (46.5)	3.2 ± 0.6	3.8 ± 0.6	< 0.001	323 (35.0)	3.4 ± 0.6	3.9 ± 0.5	< 0.001

BT: body temperature; BUN: blood urea nitrogen; IQR: interquartile range; MAP: mean arterial pressure; SCr: serum creatinine; SD: standard deviation; WBC: white blood cell.

### Model performance of external validation: three steps

First, we explored the relatedness between the development and validation datasets. The estimated discriminative ability of the membership model was demonstrated as an AUC of 0.65 (95% CI, 0.62–0.67), indicating moderate differences in case-mix and/or outcome incidence between development and validation periods. Furthermore, the two datasets differed significantly in the mean LP and its SD (development vs. validation: –2.84 vs. –2.54, P < 0.001; and 1.54 vs. 1.75, P < 0.001, respectively) (Supplementary Material 2, jocmr.elmerjournals.com).

**Figure 2:**
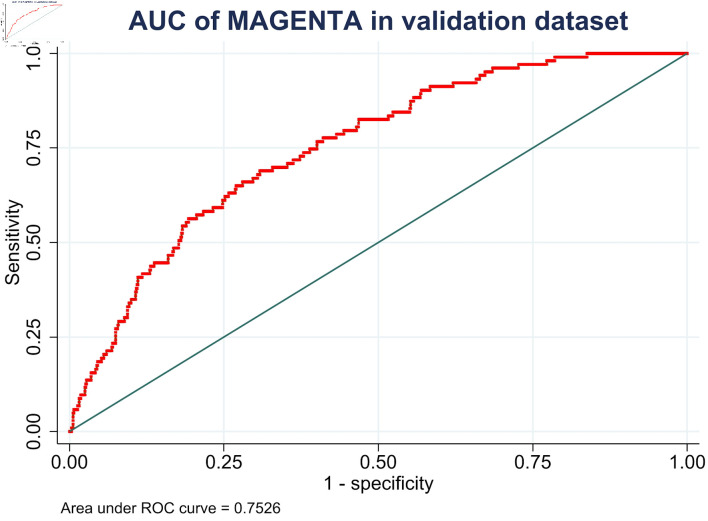
Receiver operating characteristic curve for model discrimination of the MAGENTA model in the validation cohort. AUC: area under the curve; CI: confidence interval; ROC: receiver operating characteristic.

**Figure 3:**
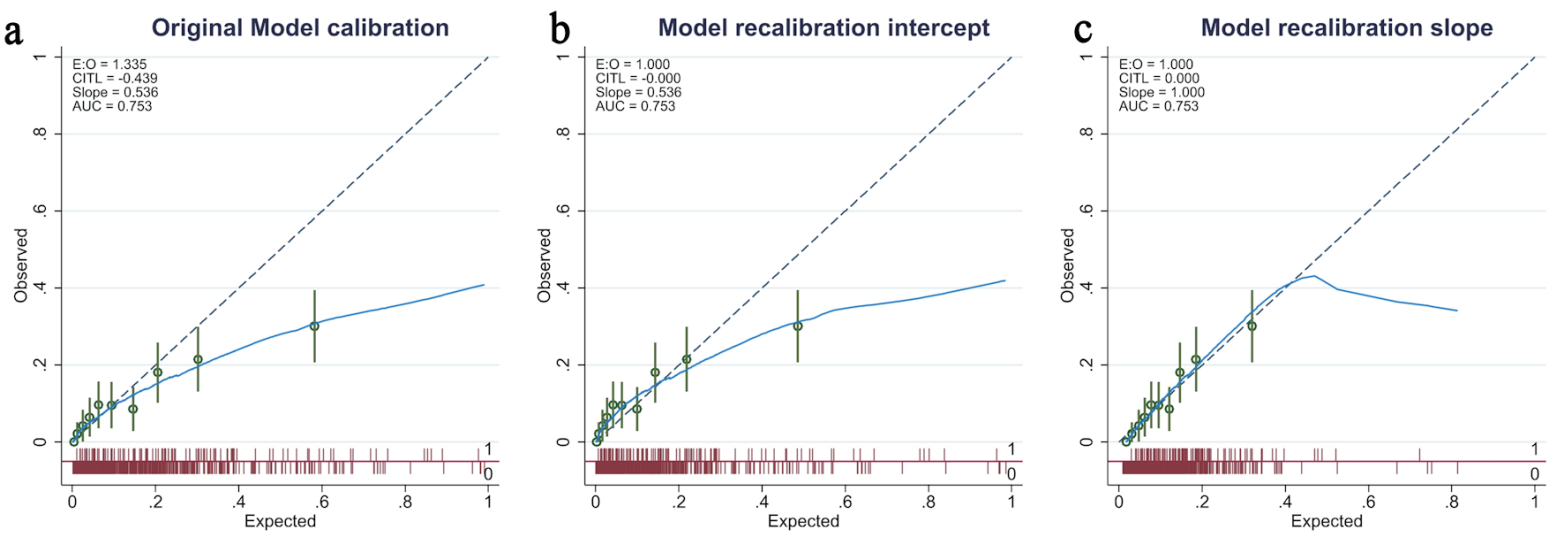
Calibration plot of the MAGENTA model in the validation cohort. (a) Original model calibration showed miscalibration, with an expected-to-observed (E:O) ratio of 1.335, calibration-in-the-large (CITL) of –0.439, and slope of 0.536. (b) After recalibration of the intercept, the E:O ratio and CITL improved to 1.000 and 0.000, respectively, although the slope remained < 1, suggesting that the prediction model was overfitting. (c) After recalibration of both the intercept and slope, calibration improved further, with E:O ratio = 1.000, CITL = 0.000, and slope = 1.000, indicating good overall agreement between predicted and observed risks.

Second, for model discrimination, the MAGENTA model showed an AUC of 0.75 (95% CI, 0.70–0.80) in the validation dataset (Fig. 2), which was lower than that in the development study at an AUC of 0.82 (95% CI, 0.77–0.86). For model calibration, the predicted probabilities of in-hospital mortality were modestly overestimated (E:O ratio = 1.335, CITL = –0.439, and calibration slope = 0.536). The calibration plot is shown in Figure 3a.

Third, based on the first step, there was evidence of a case-mix difference between the development and validation datasets. The model demonstrates acceptable performance in terms of discriminative ability. However, we observed significant systematic overprediction and evidence of overfitting, especially in patients with observed risk more than 0.2. The model updating via recalibration of the intercept and slope was provided in Figure 3b, and 3c, respectively. Details of recalibration models are provided here (Supplementary Material 3, jocmr.elmerjournals.com).

Post-hoc sensitivity analysis, in which albumin was excluded (with the coefficients set to 0), resulted in a significant drop in discriminative performance (AUC decreased from 0.75 to 0.71) in both the original and updated models (Supplementary Material 4, jocmr.elmerjournals.com). While the CITL improved from −0.439 to −0.060, the calibration slope remained unchanged in both models. Details of the results of the sensitivity analysis are presented here (Supplementary Material 4, jocmr.elmerjournals.com).

## Discussion

This study externally validated the MAGENTA model using the temporal dataset in the same setting, collected 2 years after the development of the model. The MAGENTA score consists of seven routinely available clinical predictors and showed acceptable discriminative performance in differentiating between survivor and non-survivor admissions. The MAGENTA calculator stratifies patients into three risk groups: low (< 5%), intermediate (5–15%), and high (> 15%). Patients in the low-risk group can usually be treated in general wards using standard AECOPD care pathways. These pathways include optimizing bronchodilators, controlling infections, and getting patients moving early. People at moderate risk may benefit from more frequent monitoring or step-up care, as this range often comes before clinical deterioration. Patients at high risk (> 15%) should be evaluated early for intensive monitoring or ICU admission, particularly in the presence of respiratory acidosis, ventilatory failure, or hemodynamic instability. The MAGENTA model’s performance in validation dataset exhibited acceptable discrimination (0.75 (95% CI, 0.70–0.80)), slightly lower than the previously reported 0.82 (95% CI, 0.77–0.86) [14]. However, the model substantially overestimated risk at predicted probabilities above 20%.

Variations in patients’ prognostic profiles, including predictor–outcome associations, had the potential to alter both the mean and the distribution of the model’s LPs, thereby affecting its ability to discriminate between survivors and non-survivors [19]. In this study, since the observed mortality rate in the validation cohort (11%) was comparable to that of the development cohort [14], the performance difference is likely driven by case-mix variations. This hypothesis is supported by the membership model’s AUC of 0.65, which indicates moderate heterogeneity in patient characteristics and suggests that the validation cohort was “different yet related” [19]. Beyond intrinsic patient characteristics, changes in clinical context or patient care over time, such as the increased adoption of noninvasive ventilation (NIV) during the validation period [22], may also have exerted influence. However, despite these factors, the model’s discriminative ability showed only a modest decrease. Since penalization methods were not used during development, this decline in discrimination can be partly explained by unmitigated overfitting. Regarding external calibration, the observed overestimation at predicted risks exceeding 20% is unlikely to influence clinical decisions, because patients in this range would still surpass the ICU admission threshold (> 15%). Nevertheless, this miscalibration reflects inflated absolute risks at higher probabilities, necessitating recalibration to ensure accurate risk communication.

The MAGENTA model can be a helpful bedside tool because it provides an objective measure of short-term mortality risk, thereby aiding clinicians in rapidly stratifying hospitalized AECOPD patients, especially in settings with limited ICU or monitoring resources. Risk stratification and prognostic scoring in COPD exacerbations have been advocated to tailor the intensity of monitoring, respiratory support, and therapeutic escalation [23]. The European Respiratory Society/American Thoracic Society (ERS/ATS) guideline also emphasizes that decisions about NIV, systemic steroids, and antibiotic use should be based on the severity and risk profile of the patient [11]. The strength of this model was the integration of initially available parameters upon admission, despite the unknown gap in baseline COPD-specific severity features. Furthermore, the model was externally validated in a slightly larger sample than the calculated minimum, across a wide range of clinical care settings (general ward and ICU), thereby enhancing the generalizability of the study results.

However, our study has some limitations. First, this validation dataset was derived from a retrospective cohort with missing data on serum albumin to calculate the in-hospital probability by the MAGENTA model. Although we addressed this issue by using MICE to handle missing data, the model excluding albumin resulted in a modest but statistically significant decrease in discrimination (AUC decreased from 0.75 to 0.71; P = 0.001). Second, even though there was a case-mix difference between the development and validation datasets, our study was an external model validation study in the same tertiary care center. Further geographic or broader-domain external validation studies, particularly in settings with higher use of NIV, are needed to confirm the model’s transportability before large-scale implementation. The current prediction model implies that this web-based calculator could aid clinical decision-making regarding the site of care, optimal monitoring, escalation/de-escalation of treatment, and prognostic counselling.

### Conclusions

The temporal external validation of MAGENTA for predicting in-hospital mortality among patients with AECOPD demonstrated modest reproducibility within the same setting, suggesting that it may be used as a decision-support tool in our center. However, further multicenter or broader-domain external validation across additional sites is required to confirm the model’s transportability.

## Supplementary Material

Suppl 1 Data sources and roles.

Suppl 2 Histogram of linear predictor (LP) derived from development (left column) and validation dataset (right column).

Suppl 3 Linear predictors and performance measure of the original and updated models.

Suppl 4 Predictive performance from post-hoc sensitivity analyses by excluding albumin in both original model and updated model.

## Data Availability

The authors declare that the data supporting the findings of this study are available within the article and from the corresponding author upon reasonable request.
