# Supplementary material for: The MAGENTA Model for Individual Prediction of In-Hospital Mortality in Chronic Obstructive Pulmonary Disease With Acute Exacerbation: An External Validation Study
Source: J Clin Med Res. 2026 Mar 26;18(3):196–204. doi: 10.14740/jocmr6512 (PMC13053533; doi:10.14740/jocmr6512)
Supplement: Suppl 1 — Data sources and roles. [file jocmr-18-03-196-s001.docx]

**Suppl 1.** Data Sources and Roles

| Groups | Variables | Roles | Sources |
| --- | --- | --- | --- |
| Demographic data | Gender, body weight, BMI, smoking status | Exposure | Admission record |
| Underlying diseases | Any, HT, DM, IHD, AF, LVD, CKD, CVD, CVA, cognitive impairment | Exposure | Admission record |
| COPD status | Long-term oxygen therapy, Cor pulmonale | Exposure | OPD card, Admission record |
| Spirometry result | FEV1, FVC | Exposure | Spirometry report within one year before admission |
| Medications | Any, Inhaled controller medications, influenza vaccination | Exposure | Admission record |
| Initial admission parameters | BT, HR, SBP, DBP, RR, SpO_2_ at room air | Exposure | Admission record |
| Investigation data  within 24 hours | CBC, SCr, electrolyte, glucose, chest radiography (pneumonia), endotracheal intubation | Exposure | Electronic laboratory report, Progress note |
| MAGENTA model | **M**ean arterial pressure, **A**ge, blood urea nitro**G**en, **E**ndotracheal intubation, **N**a; sodium, body **T**emperature, and serum **A**lbumin | Prediction model | Admission record, Electronic laboratory report |
| Treatment outcome | survived/ deceased | Outcome | Discharge summary |

BMI = body mass index; HT = hypertension; DM = diabetes mellitus, IHD = ischemic heart disease; AF = atrial fibrillation; LVD = left ventricular dysfunction; CKD= chronic kidney disease; CVD = cardiovascular disease; CVA = Cerebrovascular disease; FEV1 = forced expiratory volume-one second; FVC = forced vital capacity BT = body temperature; HR = heart rate; SBP = systolic blood pressure; DBP = diastolic blood pressure; RR = respiratory rate; SpO_2_ = oxygen saturation; CBC = complete blood count; SCr = serum creatinine

**Note:** All data were collected and managed using REDCap^®^ (Research Electronic Data Capture), a secure, web-based software platform designed to support data capture for research studies. An investigator, blinded to patient outcomes, performed data cleaning before the final analysis.
