# Supplementary material for: The MAGENTA Model for Individual Prediction of In-Hospital Mortality in Chronic Obstructive Pulmonary Disease With Acute Exacerbation: An External Validation Study
Source: J Clin Med Res. 2026 Mar 26;18(3):196–204. doi: 10.14740/jocmr6512 (PMC13053533; doi:10.14740/jocmr6512)
Supplement: Suppl 2 — Histogram of linear predictor (LP) derived from development (left column) and validation dataset (right column). [file jocmr-18-03-196-s002.docx]

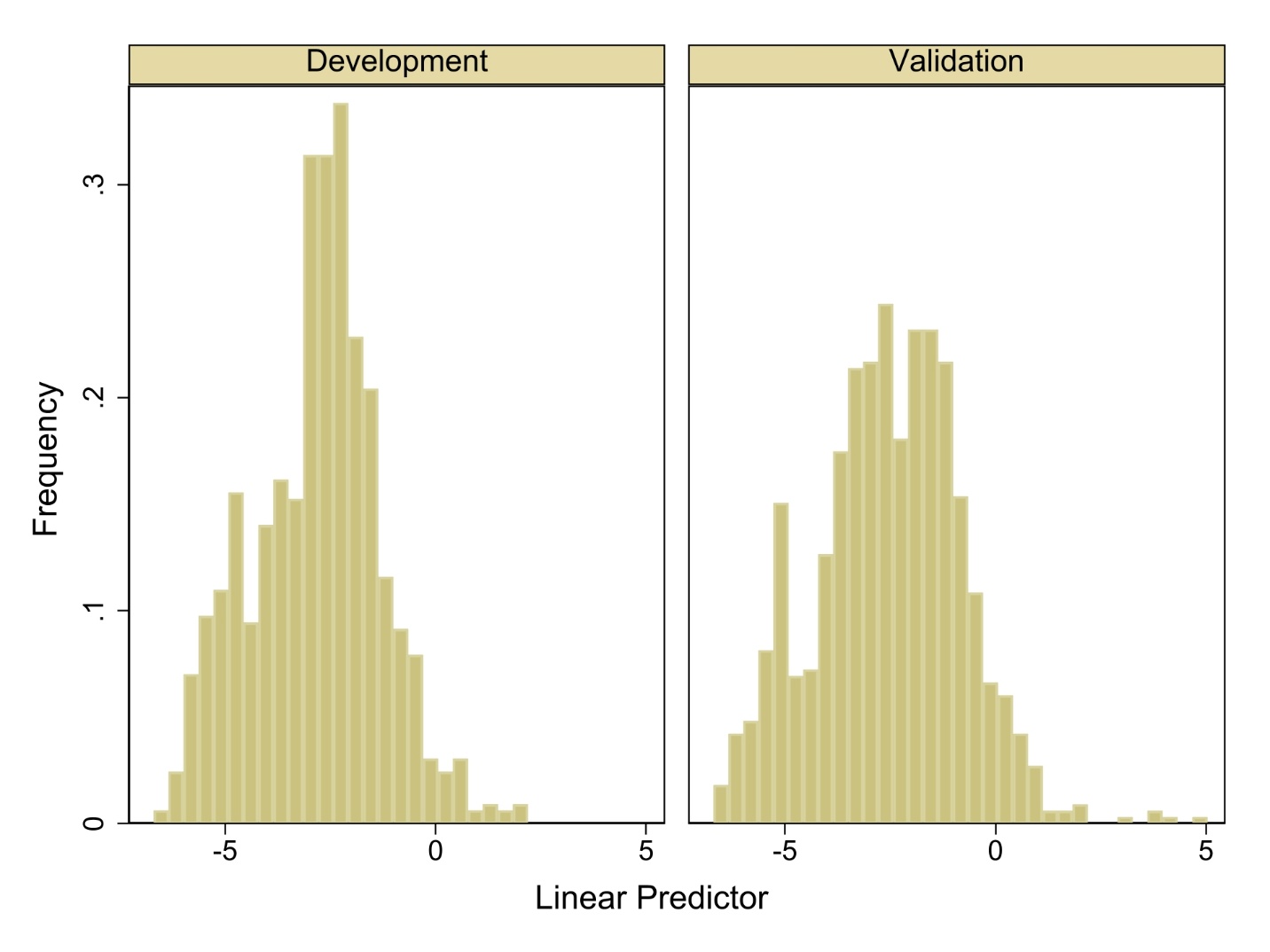


**Suppl 2.** Histogram of linear predictor (LP) derived from development (left column) and validation dataset (right column). Comparison of the mean linear predictors (LP) and its standard deviation (SD) between the development and validation cohorts. The mean LP was −2.84 in the development cohort and −2.54 in the validation cohort (p < 0.001), while the SD was 1.54 and 1.75, respectively (p < 0.001).
