# Supplementary material for: The MAGENTA Model for Individual Prediction of In-Hospital Mortality in Chronic Obstructive Pulmonary Disease With Acute Exacerbation: An External Validation Study
Source: J Clin Med Res. 2026 Mar 26;18(3):196–204. doi: 10.14740/jocmr6512 (PMC13053533; doi:10.14740/jocmr6512)
Supplement: Suppl 3 — Linear predictors and performance measure of the original and updated models. [file jocmr-18-03-196-s003.docx]

**Suppl 3.** Linear predictors and performance measure of the original and updated models

| **Predictors** | **Transformation** | **Original coefficient** | **Recalibration intercept coefficient** | **Recalibration intercept and slope coefficient** |
| --- | --- | --- | --- | --- |
| Age, years | Age-74.3651 | 0.0084 | 0.0084 | 0.0045 |
| BT | BT-37.1226 | 0.6452 | 0.6452 | 0.3459 |
| MAP | (MAP/100)^-2^-1.0545 | 0.8591 | 0.8591 | 0.4606 |
| Require intubation | Original binary form | 2.1500 | 2.1500 | 1.1528 |
| Na | Na-138.6417 | -0.0473 | -0.0473 | -0.0254 |
| BUN | BUN-18.7486 | 0.0199 | 0.0199 | 0.0107 |
| Serum albumin | Albumin-3.8174 | -1.0573 | -1.0573 | -0.5669 |
| Constant (intercept) |  | -4.4415 | -4.0022 | -2.1459 |
|  |  |  |  |  |
| AUC |  | 0.753 | 0.753 | 0.753 |
| CITL |  | -0.439 | 0.000 | 0.000 |
| Calibration slope |  | 0.536 | 0.536 | 1.000 |
|  | | | |  |

**Abbreviations:** AUC, area under the curve; BT, body temperature; BUN, blood urea nitrogen; CITL, calibration-in-the large; LP, linear predictor; MAP, mean arterial pressure; NA, sodium.
