# Supplementary material for: The MAGENTA Model for Individual Prediction of In-Hospital Mortality in Chronic Obstructive Pulmonary Disease With Acute Exacerbation: An External Validation Study
Source: J Clin Med Res. 2026 Mar 26;18(3):196–204. doi: 10.14740/jocmr6512 (PMC13053533; doi:10.14740/jocmr6512)
Supplement: Suppl 4 — Predictive performance from post-hoc sensitivity analyses by excluding albumin in both original model and updated model. [file jocmr-18-03-196-s004.docx]

**Suppl 4.** Predictive performance from post-hoc sensitivity analyses by excluding albumin in both original model and updated model.

| **Predictors** | **Original model** | **Original model**  **without albumin** | **Recalibration intercept and slope coefficient** | **Recalibration intercept and slope coefficient**  **without albumin** |
| --- | --- | --- | --- | --- |
| AUC (95% CI) | 0.75 (0.71-0.80) | 0.71 (0.67-0.76) | 0.75 (0.71-0.80) | 0.71 (0.67-0.76) |
| CITL | -0.439 | -0.060 | 0.000 | 0.000 |
| Calibration slope | 0.536 | 0.537 | 1.000 | 1.001 |
| P value for with vs without albumin model |  | 0.001 |  | 0.001 |
|  |  |  |  |  |

**Abbreviations:** AUC, area under the curve; CITL, calibration-in-the large; 95% CI, 95% confident interval.
